# SVOP Is a Nucleotide Binding Protein

**DOI:** 10.1371/journal.pone.0005315

**Published:** 2009-04-24

**Authors:** Jia Yao, Sandra M. Bajjalieh

**Affiliations:** Department of Pharmacology, University of Washington, Seattle, Washington, United States of America; Katholieke Universiteit Leuven, Belgium

## Abstract

**Background:**

Synaptic Vesicle Protein 2 (SV2) and SV2-related protein (SVOP) are transporter-like proteins that localize to neurotransmitter-containing vesicles. Both proteins share structural similarity with the major facilitator (MF) family of small molecule transporters. We recently reported that SV2 binds nucleotides, a feature that has also been reported for another MF family member, the human glucose transporter 1 (Glut1). In the case of Glut1, nucleotide binding affects transport activity. In this study, we determined if SVOP also binds nucleotides and assessed its nucleotide binding properties.

**Methodology/Principal Findings:**

We performed *in vitro* photoaffinity labeling experiments with the photoreactive ATP analogue, 8-azido-ATP[γ] biotin and purified recombinant SVOP-FLAG fusion protein. We found that SVOP is a nucleotide-binding protein, although both its substrate specificity and binding site differ from that of SV2. Within the nucleotides tested, ATP, GTP and NAD show same level of inhibition on SVOP-FLAG labeling. Dose dependent studies indicated that SVOP demonstrates the highest affinity for NAD, in contrast to SV2, which binds both NAD and ATP with equal affinity. Mapping of the binding site revealed a single region spanning transmembrane domains 9–12, which contrasts to the two binding sites in the large cytoplasmic domains in SV2A.

**Conclusions/Significance:**

SVOP is the third MF family member to be found to bind nucleotides. Given that the binding sites are unique in SVOP, SV2 and Glut1, this feature appears to have arisen separately.

## Introduction

Regulated secretion in neurons and endocrine cells is mediated by a specialized version of SNARE-mediated membrane fusion (reviewed in [1]). The unique features of regulated secretion are created, in part, by a cadre of proteins specific to neurons and endocrine cells. Among these are SV2 [2]–[6] and SVOP [7], both of which share structural similarity with the major facilitator (MF) family of small molecule transporters [7], [8].

SV2 is an essential protein that is required for normal neurotransmission [9], [10]. In neurons and endocrine cells lacking SV2, the number of vesicles capable of fusing with the plasma membrane, referred to as the readily releasable pool, is reduced [11], [12]. SV2 therefore appears to function as a modulator of vesicle priming. There are three SV2 genes in mammal that encode isoforms SV2A, SV2B and SV2C [13], [14]. All three isoforms demonstrate calcium-regulated binding to the calcium sensor synaptotagmin [15]–[17], suggesting that SV2 influences calcium-regulated priming through synaptotagmin. The recent finding that SV2 binds adenine nucleotides suggests that its action may regulate or be regulated by synaptic energy levels [18]. SV2A is the binding site of the drug levetiracetam [19], [20] and related compounds [21], which are used in the treatment of epilepsy [22] and show promise in the treatment of neuropathic pain [23], [24] and dyskinesias [25], [26]. Thus the SV2 proteins constitute a therapeutic target and are, at present, the only known drug target in synaptic vesicles.

SVOP is distantly related to SV2, sharing 20–22% sequence identity with SV2 [7]. SVOP is one of the first proteins expressed in the developing nervous system [7], [27], though beyond that very little is known about the function of SVOP, or the related protein SVOPL [28]. Although SVOP is structurally similar to SV2, it is not clear that it performs a similar function. Like SV2, SVOP co-purifies with synaptic vesicles, consistent with it being a synaptic vesicle protein. However, immunolabeling studies of brain revealed that it is also present in neuronal cell bodies [7], which is not true of SV2.

We recently reported that SV2 binds nucleotides. To determine if SVOP shares this feature of SV2, we assayed its nucleotide binding properties. Our findings indicate that SVOP is a nucleotide binding protein, but that both the specificity and binding site differ from that of SV2.

## Results

### SVOP binds 8-azido-ATP

**Figure 1:**
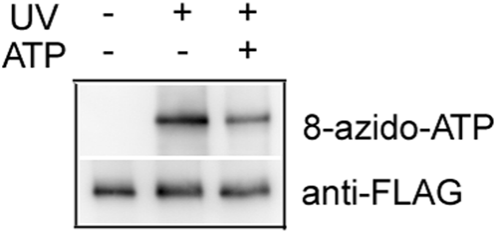
Purified SVOP-FLAG fusion proteins are labeled with 8-azido-ATP-biotin. Recombinant SVOP-FLAG fusion protein was purified from transfected HEK293 cells with Anti-FLAG M2 affinity gel. About 5 µg protein preparation was used in each photoaffinity labeling reaction with 100 µM 8-azido-ATP-biotin in the presence or absence of 1 mM non-photoreactive ATP. A control without UV photolysis was set up in parallel. The samples were resolved by SDS-PAGE and transferred to PVDF membrane for western blot analysis. The bound 8-azido-ATP was visualized by ExtrAvidin-HRP and anti-FLAG antibody was used to detect the proteins.

In a previous study [18], we found that SV2 is a nucleotide binding protein. We showed that both purified recombinant SV2-FLAG protein and native SV2 in the synaptic vesicle preparations can be labeled with the photoreactive ATP analogue, 8-azido-ATP[γ] biotin. To test whether SVOP is a nucleotide binding protein, we performed similar photoaffinity labeling experiments. Affinity-purified, recombinant SVOP-FLAG fusion protein was incubated with 8-azido-ATP[γ] biotin in the presence or absence of UV irradiation. As shown in **Figure 1**, after UV photolysis 8-azido-ATP was incorporated into recombinant SVOP-FLAG proteins. However, incorporation of the photoaffinity ATP analogue did not occur without the UV-irradiation. Furthermore, excess non-photoreactive ATP decreased the labeling. Our attempt to measure labeling of endogenous SVOP in synaptic vesicle preparation was hampered by the low abundance of SVOP in synaptic vesicles and the lack of an efficient antibody for immunoprecipitation. Given the results of SV2, however, it is likely that the photoaffinity labeling *in vitro* reflects an ability of SVOP to bind ATP *in situ*.

**Figure 2:**
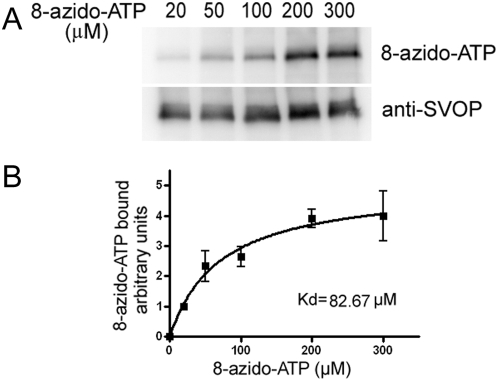
8-azido-ATP binding to SVOP-FLAG is saturable and displays a binding affinity of 83 µM. Purified SVOP-FLAG was labeled with the indicated concentrations of 8-azido-ATP-biotin and subjected to SDS-PAGE and western blot analysis. The net intensity of the regions of interest was quantified using a Kodak Image Station 440. A, Representative western blot result of SVOP-FLAG labeling as a function of 8-azido-ATP concentration. B, Quantification of the western data. The data were expressed as the intensity of 8-azido-ATP labeling normalized to SVOP protein signals. The error bars represent SEM (n = 4).

To determine the affinity of SVOP binding ATP, we measured the binding of increasing amount of 8-azido-ATP (20–300 µM). As shown in **Figure 2**, quantitative analysis of SVOP labeling showed saturation with an apparent Kd value of 83 µM, a value within physiological concentrations of ATP in cells, and similar to the apparent Kd values measured for SV2 (∼90 µM).

### ATP, NAD, and GTP produce strong inhibition in SVOP labeling

**Figure 3:**
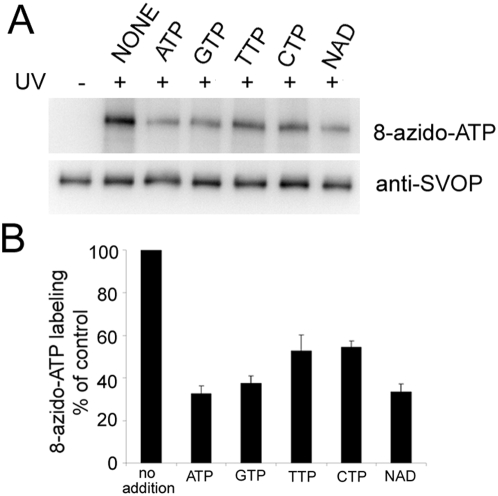
Nucleotide specificity of the 8-azido-ATP binding to SVOP. SVOP-FLAG was labeled with 100 µM 8-azido-ATP in the absence or presence of 1 mM indicated competitive nucleotides. Samples were subjected to SDS-PAGE and western blot followed by quantitative analysis. Panel A shows a representative western blot result. Panel B shows the quantification of the western blot data. The error bars represent SEM, n = 5.

To determine the specificity of nucleotide binding by SVOP, we tested the ability of 10-fold excess various nucleotides to compete with 8-azido-ATP labeling. ATP, GTP, TTP, CTP, and NAD all decreased labeling, though ATP, GTP and NAD displayed the strongest inhibition. TTP and CTP produced the least inhibition (**Figure 3**).

**Figure 4:**
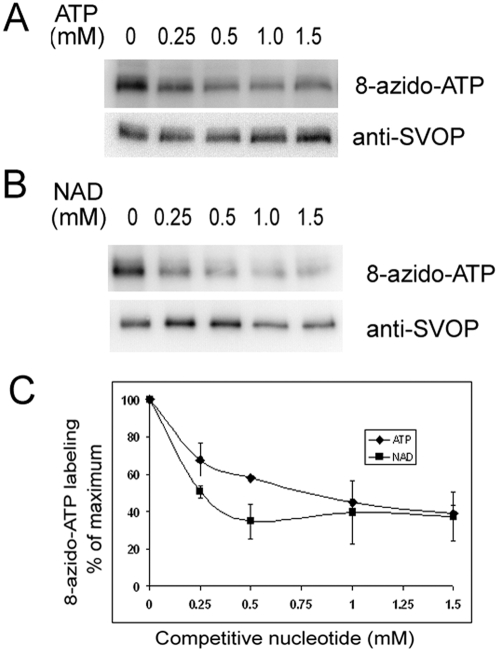
SVOP demonstrates highest affinity for NAD. SVOP-FLAG was labeled with 100 µM 8-azido-ATP in the absence or presence of ATP (0.25–1.5 mM) or NAD (0.25–1.5 mM). Data were expressed as the percentage of 8-azido-ATP labeling according to control with no ATP or NAD in the reaction. A and B were representative western blot results of SVOP labeling in the presence of different concentration of ATP (A) or NAD (B). C shows the quantification of the western blot results. Error bars represent SEM, n = 3. The half maximum inhibition concentration of NAD and ATP on SVOP ATP binding is about 0.25 mM and 0.75 mM, respectively.

To compare the affinity of SVOP for ATP and NAD, we analyzed the effects of increasing concentrations of these two nucleotides on 8-azido-ATP labeling. As shown in **Figure 4**, ATP competed with 8-azido-ATP binding to purified SVOP-FLAG with half maximum inhibition concentration of ∼0.75 mM. The concentration of NAD that produced half maximum inhibition was ∼0.25 mM. Thus SVOP appears to have the highest affinity for NAD, a feature that distinguishes it from SV2, which demonstrates equal affinity for both adenine nucleotides.

### Nucleotide binding maps to a region spanning membrane domains 9 to 12 of SVOP

**Figure 5:**
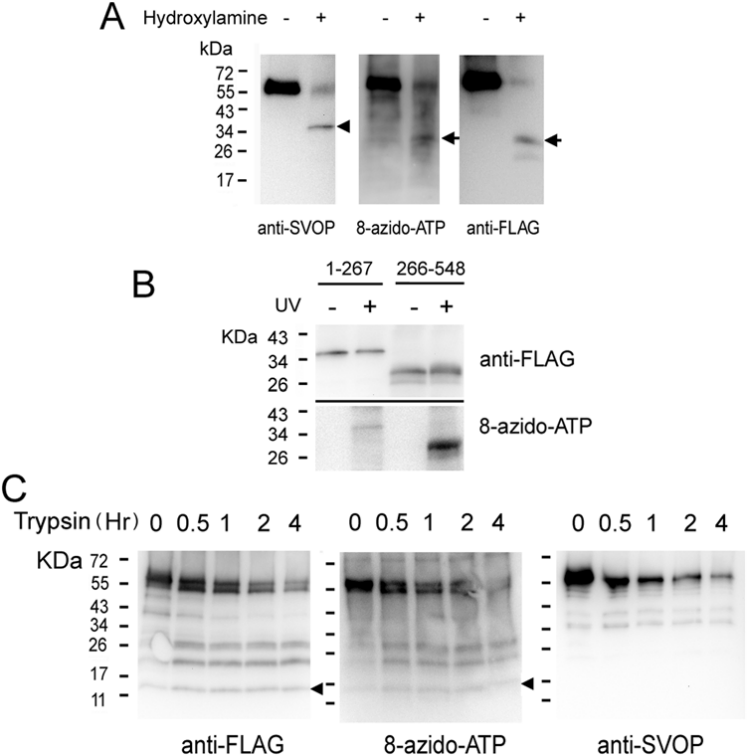
The nucleotide binding site in SVOP localizes to the C-terminal half of the protein. A. 8-azido-ATP photoaffinity labeled SVOP-FLAG was cleaved by hydroxylamine and the samples were subjected to western blot analysis using anti-FLAG, anti-SVOP and ExtrAvidin-HRP. Arrowhead indicated an N-terminal fragment which is recognized by anti-SVOP antibody but not labeled by 8-azido-ATP. The Arrows indicate a C-terminal fragment which is labeled by 8-azido-ATP and anti-FLAG antibody. B. Only the C-terminal half of SVOP shows dominant nucleotide binding. N- and C-terminal halves of SVOP-FLAG were expressed and purified from HEK293 cells. Photoaffinity labeling was performed as described under *Methods*. C. Shown are the results of trypsin digestion of 8-azido-ATP labeled SVOP-FLAG. Labeled protein was digested at 37°C in the presence of trypsin. At the time periods indicated, an aliquot was withdrawn and subjected to analysis as described under *Methods*. The arrowheads indicate the smallest trypsinized 15 kDa fragment which is labeled by both 8-azido-ATP and anti-FLAG antibody. The proportion of total anti-FLAG and 8-azido-ATP labeling (undigested protein) was similar for the 15 KDa fragment (∼9% in both cases), consistent with it being labeled with the same efficiency as the full-length protein. Therefore the nucleotide-binding site is contained within this 15 KDa fragment. The image of 8-azido-ATP labeling blot was adjusted with contrast to show better results.

To identify the nucleotide-binding site(s) in SVOP, we first applied chemical cleavage and enzymatic digestion to 8-azido-ATP-labeled SVOP-FLAG. Hydroxylamine cleaves the bond between Asn and Gly, with much less efficient cleavage at Asn-Leu, Asn-Ala, and Asn-Met bonds [29]. SVOP contains an Asn-Gly bond at residue 285–286 that is located in the cytoplasmic loop between transmembrane domains six and seven. Hydroxylamine cleavage at this bond is predicted to generate an N-terminal fragment of 31.7 KDa and a C-terminal fragment of 30.1 KDa. As shown in **Figure 5A**, hydroxylamine cleavage of SVOP-FLAG produced a fragment with an apparent molecular mass of 35 KDa that was labeled by an antibody against an N-terminal sequence in SVOP, and a fragment with an apparent molecular mass of 28.5 KDa that was recognized by an anti-FLAG antibody. The photoaffinity-labeled fragment had the same apparent molecular weight as the anti-FLAG (C-terminal) fragment. This indicates that the nucleotide-binding site in SVOP falls within the carboxyl-terminal half of the protein (residues 286–548).

We next assessed binding by recombinant proteins corresponding to the amino and carboxy halves of SVOP. Recombinant FLAG fusion proteins containing the amino (Met^1^- Val^267^) or carboxy half (Asp^266^-Glu^548^) of SVOP (denoted SVOP^1–267^ and SVOP^266–548^, respectively) were assessed for binding to 8-azido-ATP. As with hydroxylamine-cleaved SVOP, apparent molecular sizes of the FLAG tagged SVOP^1–267^ and SVOP^266–548^ in SDS-PAGE (35 and 30 kDa, respectively) differed from the calculated molecular weights of 30.9 and 32.4 kDa, respectively. Our observation that the amino portion of SVOP resolves as a larger protein in SDS-PAGE may reflect incomplete denaturation in SDS. Likewise, the apparent smaller size of carboxy end is consistent with this portion of SVOP being very hydrophobic. As shown in **Figure 5B**, the C-terminal peptide, SVOP^266–548^, bound 8-azido-ATP whereas N-terminal half protein (SVOP^1–267^ ) showed only minimal binding, confirming that the primary nucleotide binding site in SVOP is located on the carboxy terminal portion of the protein. Given that the N-terminal fragment produced by hydroxylamine cleavage did not show labeling, the very low level labeling in the N-terminal half protein is probably due to the nonspecific binding of 8-azido-ATP.

Trypsin digestion of photolabeled SVOP further defined the site of 8-azido-ATP attachment to SVOP. As shown in **Figure 5C**, trypsin digestion produced a series of small proteolytic fragments that labeled with ExtrAvidin-HRP and anti-FLAG antibody but not anti-SVOP antibody. The smallest fragment that labeled with both anti-FLAG antibody and 8-azido-ATP was ∼15 KDa, which suggests that the binding site is contained within amino acids 411–548 of SVOP. To further define the region, we generated a series of C-terminal truncations and found that SVOP lacking the last 30 amino acids (SVOP^1–518^) retained the nucleotide binding capability (data not shown). Together these data indicate that the nucleotide-binding site in SVOP is located between a.a. 411–518. We note, however, that the tendency of the carboxy end of SVOP to run smaller in SDS-PAGE suggests that the actual binding site may include residues preceding a.a. 411.

**Figure 6:**
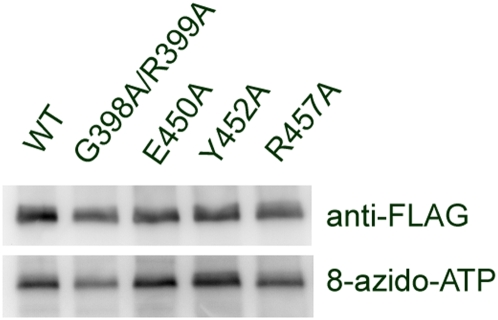
Mutants of SVOP show normal nucleotide binding. Mutated SVOP constructs were generated by site-directed mutagenesis. Photoaffinity labeling with 8-azido-ATP was performed with the wildtype and the mutated proteins. All the mutants show similar binding capability as the wild type.

The nucleotide binding sites in SV2 and another MF transporter protein, Glut1, are in cytoplamic domains. To determine if this is also true of SVOP, we mutated residues that are – conserved in SVOP across species - in the loops between membrane domains 8/9 and 10/11. SVOP-E450A, SVOP-Y425A, SVOP-R457A and the double mutant, G398A/R399A, which is analogous to residues required for nucleotide binding to Glut 1 [30], were tested for binding to 8-azido ATP. As shown in **Figure 6**, all mutant SVOP proteins demonstrated nucleotide binding similar to wild-type SVOP, indicating that the binding site does not depend on these conserved residues. Theses results also demonstrate that the ATP binding site in Glut1 is not conserved in SVOP.

## Discussion

SV2 and SVOP are transporter-like components of synaptic vesicles that share significant sequence similarity although they constitute separate protein families. SV2 proteins contain features not present in SVOP including the cytoplasmic amino terminus, the cytoplasmic loop between the sixth and seventh transmembrane domains and a luminal glycosylated loop between the seventh and eighth transmembrane domains. In the studies reported here we report that both SVOP and SV2 are nucleotide-binding proteins although both the binding site and nucleotide preference differ between the two proteins. SVOP appears to have a binding site located in a region spanning transmembrane domains 9–12, whereas SV2 has two binding sites in its large cytoplasmic domains preceding transmembrane domains 1 and 7 [18]. And whereas SVOP demonstrates the highest affinity for NAD (IC_50_∼0.25 mM versus ∼0.75 mM for ATP), SV2 shows an equal affinity for both NAD and ATP (∼0.4 mM for both).

**Figure 7:**
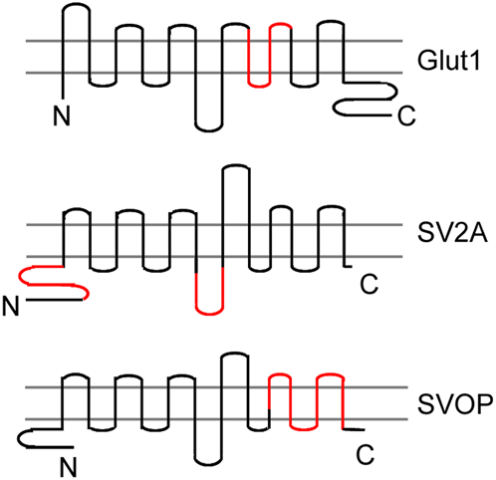
Comparison of the nucleotide binding sites in Glut1, SV2A and SVOP. Comparison of the nucleotide binding sites in Glut1, SV2A and SVOP suggests convergent evolution of nucleotide binding. Predicted 12 transmembrane domains are depicted. Red lines represent the nucleotide binding domains. The amino and carboxy termini of the proteins are indicated with letter N and C.

Nucleotide binding is also a feature of the human glucose transporter (Glut1), another member of the MF transporter family [31]. Nucleotides decrease Glut1 transport activity, thus generating an inverse relationship between transporter activity and cellular energy levels. In Glut1, the nucleotide-binding site was traced to residues 301–364 [30], [32], a region that spans membrane domains 8–9. A comparison of the binding sites in SVOP, SV2 and Glut1 is depicted in **Figure 7**, which indicate the nucleotide binding sites are different among them.

Our studies indicate that nucleotide binding is a shared feature of multiple MF transporters, though this feature appears to have arisen separately in each of the proteins. As more is learned about the molecular actions of SV2 and SVOP, it will be important to compare the effects of nucleotides on these actions to the effects of nucleotides reported for Glut1. The fact that both SV2 and SVOP demonstrate a high affinity for NAD suggests their action may be influenced by the rate of synaptic glycolysis, a process that consumes NAD, or by synaptic redox potential. It will be especially interesting to determine whether the apparent convergent evolution of nucleotide binding in MF transporters has rendered them sensitive to the same cellular conditions.

## Materials and Methods

### Plasmids

cDNA encoding rat SVOP with the FLAG epitope (DYKDDDK) fused to its C-terminus was subcloned into the mammalian expression vectors pIRES2–EGFP (Clontech, Mountain View, CA). Constructs encoding N-terminal and C-terminal halves of SVOP protein were generated by PCR amplification of rat SVOP cDNA and subcloned into FLAG-pIRES2–EGFP vector. The QuikChange Site-Directed mutagenesis Kit (Stratagene) was used to generate mutant SVOP constructs. These include single point mutations, E450A, Y452A, R457A, and a double mutation G398A/R399A.

The primers used include: E450A f 5′- TAC ACG CCT GCG GTG TAT CCA ACG GCG ACG AGG-3′, E450A r 5′- TGG ATA CAC CGC AGG CGT GTA AAC GTA GGC TGC TTG-3′, Y452A f 5′- CCT GAG GTG GCG CCA ACG GCG ACG AGG GCG-3′, Y452A r 5′- CGC CGT TGG CGC CAC CTC AGG CGT GTA AAC-3′, R457A f 5′- A ACG GCG ACG GCG GCG CTG GGC CTG GGC ACC TG-3′, R457A r 5′- GCC CAG CGC CGC CGT CGC CGT TGG ATA CAC CTC, G398A/R399A f 5′- GAC CGC CTG GCC GCC AAG AAG ACC ATG GCT CTG-3′, and G398A/R399A r 5′- GGT CTT CTT GGC GGC CAG GCG GTC GAT GAC CCA C-3′. f and r denote forward and reverse primers. Underlined letters indicate mismatches. DNA sequencing was performed to confirm that no undesired mutation was introduced by PCR.

### Cell culture and transfection

HEK293 cell culture and transfection of the cells with the Lipofectamine™ 2000 reagent were performed as previously described [18].

### Production and Purification of SVOP-FLAG protein

SVOP-FLAG fusion protein and its mutants were generated as described previously [18]. Final preparations were checked by silver staining of SDS polyacrylamide gels and immunoblot with anti-SVOP or anti-FLAG antibodies.

### Photoaffinity labeling

Dried 8-azido-ATP[γ] biotin (Affinity Labeling Technologies Inc., Lexington, KY) was dissolved in the buffer (150 mM KAc, 10 mM HEPES-KOH (pH 7.4)). Appropriate amount of 8-azido-ATP[γ] biotin as indicated was mixed with SVOP preparation and incubated on ice in the dark for 2 min. Generally, about 5 µg of SVOP-FLAG protein preparation was used for each labeling reaction and the final volume was 50 µl. After incubation, the samples were irradiated with a hand-held UV lamp at 254 nm (UVP, Inc., San Gabriel, CA) for 2 min. The UV-irradiated samples were immediately diluted with SDS-PAGE sample buffer containing β-mercaptoethanol, and subjected to SDS-PAGE and western blot analysis as described below. For hydroxylamine cleavage or proteolytic digestion experiments, the labeled samples were added with DTT to a final concentration of 40 mM after labeling. For the substrate specificity and competition studies, competitive nucleotides were added into the affinity labeling reactions.

### SDS-PAGE and immunoblotting

SDS-PAGE was performed using either precast 4%–15% gradient gels (Bio Rad Laboratories, Hercules, CA) or 10% gels. Proteins were electro-transferred onto polyvinylidene difluoride membranes (PVDF) (Millipore, Billerica, MA) and subjected to western blot analysis. The primary antibodies used include a monoclonal anti-FLAG M2 antibody (Sigma, Saint Louis, MO) and a polyclonal rabbit anti-SVOP antibody made against N-terminally derived peptides of SVOP (LRQLPVVKFRRTGES). Membranes were then incubated with the appropriate horseradish peroxidase (HRP) conjugated secondary antibody, washed and immune complexes were visualized using an enhanced chemiluminescence detection kit (Pierce, Rockford, IL). The 8-azido-ATP[γ] biotin labeled samples were probed with ExtrAvidin-HRP (Sigma, Saint Louis, MO) and visualized by chemiluminescence. The net intensity of the regions of interest was quantified using a Kodak Image Station 440, and only the nonsaturated images were used for quantification analysis. The image of 8-azido-ATP labeling blot with trypsin digested SVOP-FLAG (**Figure 5C**) was adjusted with contrast to show better results.

### Hydroxylamine cleavage of photolabeled protein

5–10 µg of 8-azido-ATP[γ] biotin labeled SVOP- FLAG protein was mixed with equal volume of hydroxylamine cleavage reaction buffer (3M hydroxylamine-HCl, 3M guanidine-HCl, adjusted to pH 9.0 with NaOH) and incubated at 45°C for 4 h. The cleavage reaction samples were then desalted by passing through Zeba desalt spin columns (Pierce, Rockford, IL). Aliquots of non-cleaved and cleaved samples were resolved by SDS-PAGE and subjected to western blot analysis with anti-FLAG, anti-SVOP and ExtrAvidin-HRP as described above.

### Proteolytic digestion of photolabeled protein

8-azido-ATP[γ] biotin labeled SVOP- FLAG protein was digested at 37°C in the presence of sequencing grade trypsin (30∶1 protein: enzyme by weight) (Boehringer Mannheim, Mannheim, Germany). At the time periods indicated, an aliquot was withdrawn and the digestion terminated by adding phenylmethylsulfonyl fluoride (PMSF) to a final concentration of 1 mM. The samples were subjected to SDS-PAGE and western blot analysis.
